# Supplementary material for: Clinical Outcomes of Different Calcified Culprit Plaques in Patients with Acute Coronary Syndrome
Source: J Clin Med. 2022 Jul 11;11(14):4018. doi: 10.3390/jcm11144018 (PMC9316434; doi:10.3390/jcm11144018)
Supplement: Supplementary file 1 [file jcm-11-04018-s001.zip › jcm-1769200-supplementary.pdf]

Supplementary Material

Supplementary Methods

1. **Diagnosis of acute coronary syndrome (ACS)**

The current American Heart Association (AHA)/American College of Cardiology (ACC) guidelines were used to make the diagnosis of ACS, which includes ST-segment elevation myocardial infarction (STEMI) and non–ST-segment elevation acute coronary syndromes (NSTE-ACS) [1, 2]. STEMI was defined as continuous chest pain that lasted >30 min, arrival at the hospital within 12 h from the onset of symptoms, ST-segment elevation >0.1 mV in ≥2 contiguous leads or new left bundle-branch block on the 12-lead electrocardiogram, and elevated cardiac markers (creatine kinase-MB or troponin I) [1]. NSTE-ACS included non–ST-segment elevation myocardial infarction (NSTEMI) and unstable angina pectoris (UAP). NSTEMI was defined as ischemic symptoms in the absence of ST-segment elevation on the electrocardiogram with elevated cardiac markers. UAP was defined as having newly developed/accelerating chest symptoms on exertion or rest angina within 2 weeks without biomarker release [2].

2. **The measurement of calcification**

The calcification arc, minimal calcification depth, and maximal calcification thickness were evaluated in each cross-sectional view, and the average values were calculated, respectively [3]. Measuring the length of the calcification in the longitudinal view. The calcification index was defined as the product of the average calcification arc and the calcification length.

3. Definitions of microstructures

Microchannels were defined as signal-poor vesicular or tubular structures delineated in multiple contiguous frames. Thrombus was defined as an irregular mass with a minimal diameter of 250 μm attached to the wall of a blood vessel or floating in the lumen. There are three types of thrombus: (a) red thrombus (high backscattering with high signal attenuation); (b) white thrombus (homogenous backscattering with low signal attenuation); or (c) mixed thrombus (features observed in both red and white thrombi). Macrophage accumulation was defined as signal-rich, distinct or confluent punctuated regions with heterogeneous backward shadowing. Cholesterol crystals were recorded as thin, linear regions of high intensity, usually associated with a fibrous cap or necrotic core. Layered plaque phenotype was defined as a plaque with heterogeneous signal-rich layered tissue of different optical signal intensities located close to the luminal surface that was clearly demarcated from the underlying tissue. The maximal area and thickness of the layered plaque were determined by the cross-sectional OCT image.

4. Post-procedure analysis of calcified plaque characteristics

Stent edge dissection (SED), in-stent dissection, calcium fracture, incomplete stent apposition (ISA), in-stent tissue protrusion, minimal stent area, stent under-expansion, and stent eccentricity were evaluated using postprocedural OCT imaging data [4]. SED was defined as disruption of the vessel luminal surface with a visible flap at the stent edge or within 5-mm proximal or distal reference segments. In-stent dissection was defined as disruption of the luminal vessel surface within the stented segment. Calcium fracture was characterized by a

calcium gap and direct calcium exposure to the lumen. ISA was evaluated by the separation of a stent strut from the vessel wall in a segment without a side branch. It was defined as a measured distance greater than the strut thickness for a bare-metal stent or greater than the sum of the thicknesses of the strut and polymer for a drug-eluting stent. In-stent tissue protrusion was classified into the following three types: (a) smooth protrusion (bowling of a plaque into the lumen between struts, appearing as a smooth convex semicircular arc); (b) disrupted fibrous tissue protrusion (underlying fibrous tissue disruption protruding between struts); and (c) irregular protrusion (material with an irregular surface protruding into the lumen between struts). The postprocedural mean reference lumen area was defined as the mean of the largest lumen area within 5-mm of the proximal and distal stent edges. The stent expansion ratio was defined as the ratio of the MSA to the postprocedural mean reference area. Stent under-expansion was defined as a stent expansion ratio < 0.8. The eccentricity of the stent was calculated as [(maximal stent diameter – minimal stent diameter)/maximal stent diameter]. The Inter- and intra-observer kappa coefficients for superficial calcific sheets were 0.91 and 0.94; eruptive calcified nodules were 0.93 and 0.95; and calcified protrusion was 0.93 and 0.92.

5. Definitions of Clinical Outcomes

Cardiac death was defined as death in the presence of ACS, significant cardiac arrhythmia, or refractory congestive heart failure, or death attributed to cardiovascular cause at post-mortem; Target vessel myocardial infarction was diagnosed according to the 4th universal definition of MI [5]. Which was defined as typical chest pain accompanied by a rise of more than two times the upper reference limit of troponins, development of new Q waves on the ECG, or both;

Ischemia-driven target lesion revascularization was defined as either percutaneous or surgical revascularization at the culprit lesion site identified at index procedure for angina or angina equivalent symptoms. Stroke was defined by the presence of neurological dysfunction and vascular brain injury caused by cerebral ischemia or hemorrhage [6]. Major bleeding was defined as any fatal bleed, intracranial bleed, intrapericardial bleed with cardiac tamponade, or hypovolemic shock/severe hypotension caused by bleeding requiring pressor or surgery; a fall in hemoglobin of ≥ 5 g/dL; or a need for transfusion of ≥ 4 units red cell concentrates. Other major bleeding was defined as significantly disabling bleeding, a fall in hemoglobin of ≥ 3 g/dL but <5 g/dl, or a need for transfusion of at least 2 units of red cell concentrates. The clinical status and quality of life after acute myocardial infarction were reflected in hospitalization for UAP. Specifically, UAP was diagnosed if the symptoms worsened with an increase in severity or length of anginal attacks.

References

1. O'Gara PT, Kushner FG, Ascheim DD, et al. 2013 ACCF/AHA guideline for the management of ST-elevation myocardial infarction: a report of the American College of Cardiology Foundation/American Heart Association Task Force on Practice Guidelines. *Circulation* 2013; 127: e362-425.

2. Amsterdam EA, Wenger NK, Brindis RG,, et al. 2014 AHA/ACC guideline for the management of patients with non-ST-elevation acute coronary syndromes: a report of the American College of Cardiology/American Heart Association Task Force on Practice Guidelines. *Circulation* 2014; 130: e344-426.

3. Wang X, Matsumura M, Mintz GS, et al. In Vivo Calcium Detection by Comparing Optical

Coherence Tomography, Intravascular Ultrasound, and Angiography. *JACC Cardiovasc Imaging* 2017; 10: 869-879.

4. Soeda T, Uemura S, Park SJ, et al. Incidence and Clinical Significance of Poststent Optical Coherence Tomography Findings: One-Year Follow-Up Study From a Multicenter Registry. *Circulation* 2015; 132: 1020-9.

5. Thygesen K, Alpert JS, Jaffe AS, et al. Fourth Universal Definition of Myocardial Infarction (2018). *J Am Coll Cardiol* 2018; 72: 2231-2264.

6. Hicks KA, Mahaffey KW, Mehran R, et al. 2017 Cardiovascular and Stroke Endpoint Definitions for Clinical Trials. *J Am Coll Cardiol* 2018; 71: 1021-1034.
